# Evaluation of the association between excessive screen use, sleep patterns and behavioral and cognitive aspects in preschool population. A systematic review

**DOI:** 10.1007/s00787-024-02430-w

**Published:** 2024-04-02

**Authors:** Llanos Merín, Abel Toledano-González, Luz Fernández-Aguilar, Marta Nieto, Nuria del Olmo, José M. Latorre

**Affiliations:** 1 Department of Psychology, Faculty of Education, University of Castilla-La Mancha, Albacete, Spain; 2 Department of Psychology, Faculty of Health Sciences, University of Castilla-La Mancha, Talavera de la Reina, Spain; 3 Biomedicine Institute, University of Castilla La Mancha, Albacete, Spain; 4 Department of Psychology, Faculty of Law, University of Castilla-La Mancha, Albacete, Spain; 5 Department of Psychology, Faculty of Medicine, University of Castilla-La Mancha, Albacete, Spain; 6 Department of Psychobiology, Faculty of Psychology, National University of Distance Education, Madrid, Spain

**Keywords:** Preschoolers, Sleep, Screen use, Externalizing, Internalizing, Cognition

## Abstract

In this review, we analyzed the possible relationship between the excessive use of screens and sleep patterns, and how this may affect certain behavioral and cognitive factors in preschool children. The selection, extraction and synthesis of the data were conducted according to PRISMA guidelines. The search was carried out in the electronic databases Medline (PubMed), PsycINFO (American Psychological Association), Scopus and Web of Science (WOS). Of the 597 articles initially identified, 13 met the inclusion criteria. The risk of bias of the articles selected was evaluated using a specific scale created for this purpose. The results found indicate that excessive use of screens is associated with a negative impact on the duration and quality of sleep-in preschoolers, and this worsening of sleep in infancy is related with a greater probability of the appearance of internalizing and externalizing behavioral problems and certain cognitive problems. The results also suggest that sleep could play a mediating or moderating role as a bioregulatory system that attenuates or increases the onset of behavioral and cognitive difficulties in those children most exposed to digital devices.

In recent years, there has been a rapid growth in new digital technology, and, despite its advantages, excessive digital media use by children has been associated with physical and psychological adverse health consequences [1]. This line of research has paid particular attention to how the use of digital devices may affect young children’s sleep [2]. Sleep is a basic physiological process characterized by intense brain activity. It promotes physical and mental well-being and has an impact on multiple aspects of development from early childhood [3]. Indeed, sleep problems in early childhood are associated with a number of poor developmental outcomes in neurocognitive [3–5], socioemotional [6, 7], physical health [8, 9] and family functioning [10–12].

Most sleep disorders in childhood may be attributed to variations in one or more of its basic parameters [6]. According to Buysse [13] and Meltzer et al. [14], six main parameters can be identified: (1) sleep duration; (2) efficiency; (3) time spent sleeping; (4) alertness during the day; (5) subjective quality; and (6) behaviors associated with sleep, including screen use. The relationship between these sleep parameters and different health risks from early childhood onwards has been the subject of a large body of research that concludes there is a bidirectional relationship between sleep disorders and physical and psychological problems [6, 15]. However, much of the research has focused on adult populations or on school-age children and adolescents, with data on preschoolers being scant and inconclusive [16].

## Sleep measurement and development in preschoolers

Although studies with preschoolers typically use sleep diaries or parent-report questionnaires, as they are easy to use and inexpensive and have shown to be useful for estimating some aspects of children’s sleep, these types of subjective sleep measures are susceptible to the biases of those who complete them, such as inaccuracies in recall or social desirability bias [17, 18]. However, the use of objective measures, such as actigraphy or polysomnography (PSG), may be useful in overcoming these limitations. In this sense, the most complete test for the study of sleep and its phases is PSG [19], although the costs associated with the use of PSG and the laboratory context in which it is implemented hinder its application in studies using preschool samples [20]. For this reason, several studies have resorted to the use of actigraphy as a reference method for estimating sleep in everyday contexts since the algorithms applied have high precision and sensitivity [21, 22]. Actigraphy is a non-invasive technique that permits objective estimation of sleep parameters by recording the frequency of movement in terms of acceleration. Its results tend to be highly consistent with data provided by parental sleep reports [23].

Sleep duration, quality and architecture change across the lifespan, especially in the first five years of life, given that the process is dependent on the maturation of the central nervous system, the environment and experience [24]. The most important changes occur from birth to age two. These are due, in part, to the processes of brain maturation, neuroplasticity and myelination that take place in this developmental period [25]. From the second year and throughout the preschool stage, changes in sleep are more gradual [26, 27]: the total number of hours of sleep progressively decreases and most of this sleep takes place during the night, with 10–13 h of sleep being recommended; daytime naps decrease; the amount of REM sleep is reduced; the length of REM-NREM cycles extends to 90 min; sleep onset latency (i.e., the time it takes the child to fall asleep after going to bed) increases; and fewer awakenings occur, thus improving sleep efficiency. From the age of 6, sleep structure begins to resemble that of adults [28].

## Screen use in childhood and its effect on sleep

Guidelines published by prominent institutions recommend avoiding screen use until the age of two, and reducing such use in preschoolers to between 30 min and an hour a day [29, 30]. However, the burgeoning role of digital devices in the 21st century and their presence in children’s lives from birth complicates complying with these recommendations.

Excessive time using digital devices can influence sleep disturbances in preschoolers in several ways: (1) electromagnetic radiation, perceived as the blue light from screens, can affect melatonin levels and, consequently, cause circadian disruption and sleep problems [31, 32]; (2) the length and time of screen use can be detrimental to sleep time or the performance of other activities that help acquire good sleep habits, thus impairing sleep quantity and quality [33, 34]; and (3) the type of content accessed, given that exposure to violence or adult content elevates a child’s psychophysiological arousal, affecting pre-sleep relaxation and increasing the likelihood of delayed sleep onset and disruptions in sleep continuity [33, 35, 36].

## Sleep and behavioural problems in preschoolers

Sleep disturbances in childhood tend to be comorbid with behavioral disorders [6, 37], especially in terms of internalizing and externalizing problems. The former have been associated with inwardly directed behaviors and emotional states, such as withdrawal, anxiety, inhibition or mood instability, while externalizing symptoms refer to outwardly directed behaviors and mood states and are characterized by a lack of emotional regulation, manifest in behaviors such as anger and irritability, aggression, hyperactivity or rule breaking [38].

Previous studies have reported an association between sleep problems and a higher level of externalizing and internalizing symptoms. For example, Scharf et al. [39] examined, using maternal reports, how nighttime sleep duration affected externalizing behavior in 4-year-olds, concluding that shorter sleep duration was associated with a greater likelihood of hyperactivity, aggressiveness, impulsivity, and tantrums. Likewise, relationships have been found with internalizing symptoms, such as emotional difficulties, anxiety or somatization [40, 41]. Similar results were obtained with 2-to 4-year-olds years in a longitudinal study, with longer sleep duration and sleep quality being predictors of fewer behavioral problems [42]. Furthermore, few works have used using actigraphy in preschool population are their results are inconsistent: while some suggest significant links between sleep problems and aggressive behaviors and emotional problems (e.g., [43]), others report no significant relationships (e.g., [44]).

## Sleep and cognition in preschoolers

The first years of life are a fundamental stage for cognitive development, including aspects such as memory, language, and executive functions [45]. However, despite the growing evidence on the importance of sleep for proper cognitive functioning in preschoolers [15, 46], most studies have focused on school-aged children [47 for a review]. with information on infant and preschool samples continuing to be scant. Additionally, considering that the characteristics and architecture of sleep change rapidly during this life stage, findings from studies on older participants cannot reasonably be generalized to younger children [48].

Cross-sectional studies with preschoolers have yielded contrasting data. The work by Nathanson and Fries [49] found no relationship between parent-reported sleep time and performance in inhibition, working memory, and cognitive flexibility. More recently, the study by Peled and Scher [50], with a sample of preschoolers aged 3 to 4 years, showed an association between working memory scores and sleep quality, but not with duration. Finally, Nieto et al. [5], with a sample of 3- to 6-year- olds, found that parent-reported nighttime sleep duration was related to inhibition and working memory, but not to cognitive flexibility. In addition, longitudinal data on preschoolers seem to indicate that children who present correct sleep development from very early ages tend to show better performance in subsequent stages (e.g., [8, 21]).

Given the importance of sleep in certain behavioral and cognitive processes, and knowing screen use can affect sleep patterns, we can hypothesize that excessive screen use at preschool age negatively affects behavior and cognition in the youngest children. For this review, we consider as excessive use of screens the one which exceeds the recommendations established by the health authorities, that is, more than an hour for preschool-age children [27, 28]. Hence, the main aim of this work was to systematically analyze the empirical evidence on the relationships between excessive screen use and sleep patterns in 3- to-6-olds years, as well as the impacts on psychological (internalizing and externalizing problems) and cognitive functioning.

To achieve this general aim, the following specific objectives were proposed: (a) to determine the impact of excessive screen use on sleep at the preschool stage; (b) to detect how these aspects related to sleep may impact different behavioral and cognitive factors; (c) to ascertain whether excessive screen use may influence the emergence of behavioral and cognitive problems in preschool years; and (d) to evaluate the findings of the contributions and identify future research directions.

## Materials and methods

This study used the criteria for conducting systematic reviews described by the PRISMA methodology [25]. To formulate the research question, the following format was used: problem of interest, population, exposure and outcome. The resulting question was thus as follows What is the relationship between excessive screen use, sleep-related aspects, and the appearance of psychological and/or cognitive effects in preschool children (3–6 years)? To answer this question and limit the search to the topic of interest, the descriptors for health sciences (DeCS) were used, with English always being the working language.

### Search strategy

An exhaustive electronic search of the Medline (PubMed), PsycINFO (American Psychological Association), Scopus and Web of Science databases was conducted from February 7 to April 30, 2022. This search was applied to the fields Title + Summary + Keywords. The terms included in the search were “preschool” OR “early childhood” OR child*; -AND “screen time” OR “screen media” OR “media exposure” OR “television” OR “electronic device” OR “tablet” OR “mobile” OR “technology” -AND “sleep duration” OR “sleep disturbance” OR “sleep disorder” OR “sleep quality” -AND “behavior” OR externali* OR internali* OR cognit*. An analysis of the bibliographic references of the included studies completed the electronic search.

### Inclusion and exclusion criteria

In accordance with the aims of the present study, we selected publications meeting the following inclusion criteria: (a) the study sample had to consist of preschoolers aged between 3 and 6; (b) the study included measures of sleep duration, reported by primary caregivers or actigraphy; other primary caregiver self-report measures referring to sleep patterns could also be included; (c) screen exposure measures necessarily included time of use; (d) externalizing and/or internalizing behaviors were measured using validated questionnaires for participants or primary caregivers; and (e) cognitive variables focused on language, executive functions and/or theory of mind, and were measured by questionnaires administered by an expert. The following were excluded: (a) studies published in a language other than English; (b) studies with samples of preschoolers with psychopathologies or learning disabilities; (c) studies without measurements of any of the variables of interest (sleep, screen exposure and behavior/cognition), or where such measures could not be estimated with the data reported, or those that did not establish relationships between these variables; (d) intervention studies; or (e) theoretical or systematic reviews, meta-analyses, theses or book chapters.

### Data extraction and analysis

**Fig. 1:**
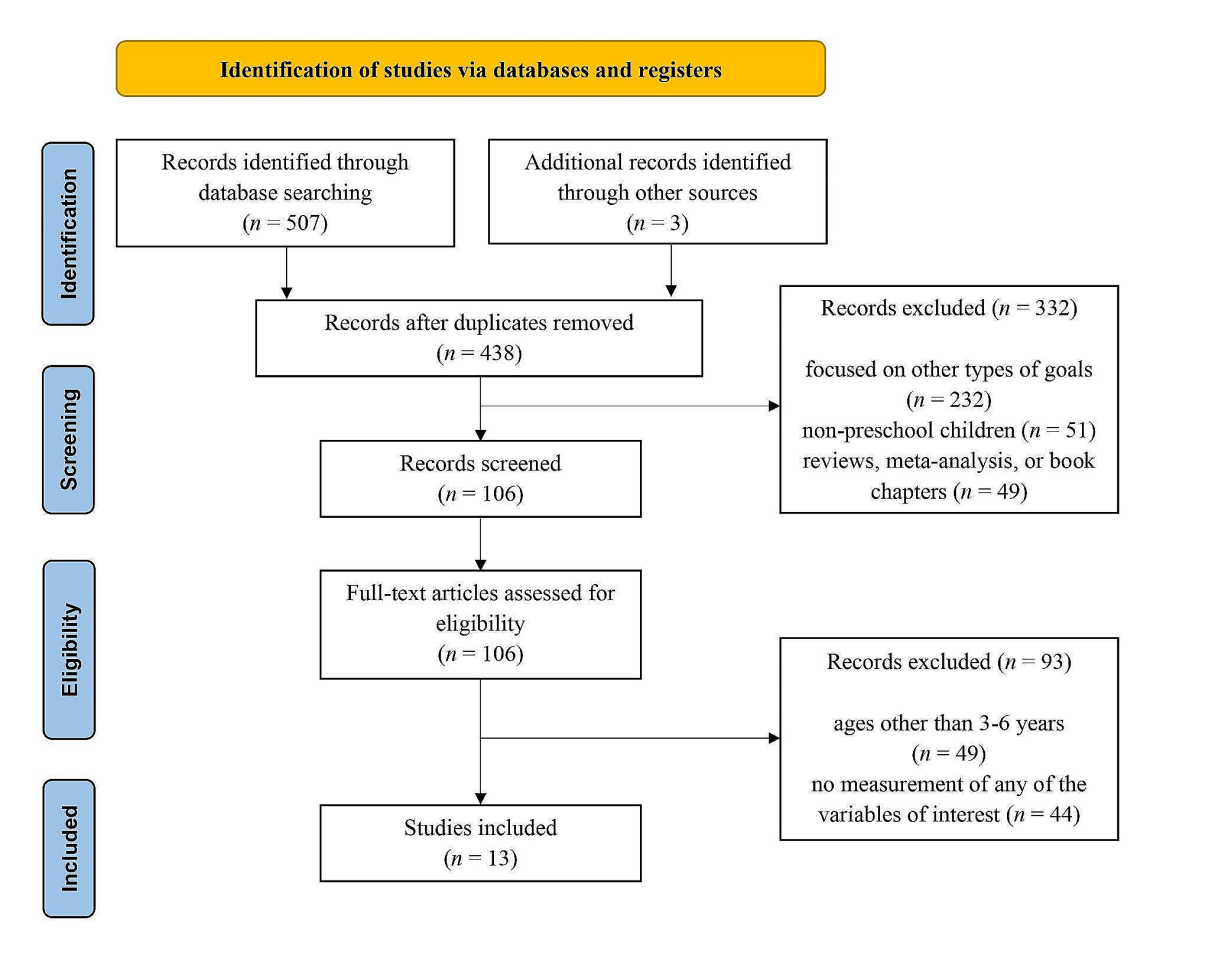
PRISMA flow diagram

Following the initial bibliographic search, duplicates were excluded from the selection of articles identified. Subsequently, we reviewed the titles and abstracts of the remaining articles to check they met the inclusion and exclusion criteria, and those eligible for inclusion were selected. Articles that raised doubts were evaluated by an independent reviewer until a decision was reached on their inclusion or exclusion (see Fig. 1 for a flow chart adapted from the PRISMA methodology).

### Risk of bias

The quality of the included articles was assessed using a list of methodological criteria related to the topic of interest (See Table 1). The criteria used to carry out the quality analysis were established based on the Newcastle-Ottawa Scale (NOS) for studies that are not clinical trials [51]. Twelve criteria were used: (1) sociodemographic data of the sample were described (age, race, parents’ educational level, etc.); (2) inclusion/exclusion criteria were included; (3) the data collection process was described (e.g., interview, parental report); (4) the study sample was randomly selected; (5) the sample size was greater than 500; (6) comparisons were made between groups based on exposure variables (sleep and screen use); (7) the “screen use” variable was objectively evaluated; (8) the “sleep” variable was objectively assessed by actigraphy; (9) standardized, validated questionnaires were used for behavioral and cognitive variables; (10) behavioral and cognitive variables were assessed by an individual blinded to exposure status; (11) statistical adjustments were made to account for the main confounding factors; and (12) the results were followed up. Each criterion was scored as met/not met. The total study quality score was obtained from the sum of the criteria met, with scores ranging from 0 to 12. Study quality was rated as low (0–4), medium (5–8), or high (9–12).

**Table 1:** List of criteria for assessing the methodological quality of studies

	Carson, 2019 [52]	Cliff, 2017 [53]	Helm, 2019 [54]	Kahn, 2021 [55]	McNeill, 2020 [56]	Mistry, 2007 [57]	Nathanson, 2018 [58]	Nathanson, 2015 [49]	Séguin, 2016 [59]	Tso, 2016 [60]	Wu. 2017 [61]	Zhao, 2022 [62]	Zhao, 2018 [63]
1. The sociodemographic data of the sample (age, race, parents’ educational level, etc.) are described.	X		X	X	X	X	X	X	X	X	X	X	X
2. The inclusion/exclusion criteria are included.	X	X	X	X			X					X	
3. The data collection procedure is described (e.g., interview, parental report).	X	X	X	X	X	X	X	X	X	X	X	X	X
4. The sample is randomly selected.	X	X	X	X	X	X	X	X	X	X	X	X	X
5. Sample size is larger than 500.	X									X	X	X	X
6. Comparative analyses are conducted between groups according to exposure variables (sleep and screen use).	X	X	X	X	X	X	X	X		X	X	X	X
7. The “screen use” variable is o measured objectively.													
8. The “sleep” variable is evaluated objectively (actigraphy).	X		X	X									
9. Standardized and validated questionnaires are used for the behavioral and cognitive variables.	X	X	X	X	X	X	X	X	X	X	X	X	X
10. The behavioral and cognitive variables are assessed by a researcher blind to the exposure status (screen time and sleep).		X			X			X	X	X			
11. Statistical adjustments are made to take the main confounding factors into account.	X	X		X	X	X	X	X		X	X	X	X
12. The results are followed up (longitudinal approach).					X								
**Total score**	**9**	**7**	**7**	**8**	**8**	**6**	**7**	**7**	**5**	**8**	**7**	**8**	**7**

## Results

A total of 510 studies were identified. Of these, 72 were dropped due to their being duplicates. Of the remaining 438, titles and abstracts were reviewed, and 332 were excluded (232 focused on a topic other than that of interest; 51 included samples of non-preschoolers; and 49 were review studies, meta-analyses or book chapters). This left 106 texts to be read in full. Of these 106 studies, 49 included populations of the wrong age range and 44 did not measure one or more of the variables of interest considered in the present review. Finally, 13 articles were finally selected for this systematic review (Table 2).

### Characteristics of the studies included

The scores for the quality of the studies included ranged from 5 to 9 points The mean quality was 7.2 points, meaning the overall quality of the results obtained is medium. The methodological deficiencies are mainly related to the lack of standardized questionnaires to evaluate screen use in preschool population, the absence of follow-up of the results obtained, and the use of subjective sleep reported through questionnaires completed by the children’s parents or caregivers.

All the studies used a cross-sectional design, and only the work by McNeill et al. [56] included a 12-month longitudinal follow-up. The studies were published between 2007 and 2022.

Sleep time was assessed in various ways: 46.2% of the studies included only direct parental reports (*n* = 6), 30.8% used parent-completed questionnaires (*n* = 4), and 15.4% used only actigraphy (*n* = 2); the remaining study [55] used actigraphy together with parental reports. The participants’ mean nighttime sleep duration ranged from 9.15 h/d [61] to 10.6 h/d [58]. Other measures of sleep patterns were sleep onset latency, night wakings, efficiency, bedtime resistance, sleep anxiety, parasomnias and daytime sleepiness. All the studies measured screen time by means of direct parent reports consisting of open-ended questions (e.g., “On average, how many hours per day does your child sit and watch TV?“), given the absence of standardized measures. Regarding the types of digital devices, two focused exclusively on TV viewing [54, 57], while the rest explored multiple devices, with the most common being TV (*n* = 11), computers (*n* = 9), and tablets (*n* = 7). The studies primarily recorded mean active TV viewing time, which ranged from 2.8 h/d [61] to 3.8 h/d [58]. Other aspects of screen use taken into account by the researchers were passive exposure, presence of screens in the child’s room or proximity of use to sleep periods.

Finally, as regards behavioral and cognitive aspects, most of the studies focus on the analysis of internalizing and externalizing behaviors (*n* = 11). Those incorporating cognitive measures mainly implemented measures of performance for Theory of Mind (*n* = 5) and Executive Functions (*n* = 3).

**Table 2:** Overview of studies included in the review

Study	Study Quality	Participants	Country	Design	Measures and Instruments	Results	General Conclusions
Carson et al. (2019) [52]	9	*N* = 539 3 years 47.9% Female 52.1% Male	Canada	Cross-sectional	Sleep: actigraphy Screen: parent report Internalizing /Externalizing: CBCL	50.5% and 83.1% of children met the screen time and sleep recommendations, respectively; 41.7% met both. Children not meeting screen time recommendations had a significantly high total problem score (B = 3.04, *p* < 0.001), externalizing score (B = 2.72, *p* = 0.001) and internalizing score (B = 1.94, *p* = 0.022) Meeting recommendations for the combination of sleep and screen time was associated with a lower score of total problems (B = 2.31, *p* = 0.006) and externalizing problems (B = 2.14, *p* = 0.046), but not with internalizing problems (B = 0.85, *p* = 0.326).	Less than half the sample complied with both recommendations on screen use and sleep time. Meeting both recommendations was associated with fewer behavioral and emotional problems, as measured by the CBCL. Associations were strongest for total and externalizing problems.
Cliff et al. (2017) [53]	7	*N* = 248 3–6 years (x̄ = 4.2, *SD* = 0.6) 43.2% Female 56.8% Male	Australia	Cross-sectional	Sleep: parent report Screen: parent report Cognition: TEC ToM Scale	Screen use (min/d): *M* = 139.8, *SD* = 83.4; Sleep (hrs/d): *M* = 10.5, *SD* = 1.0. 17.3% and 88.7% of children met the screen time and sleep guidelines, respectively. Children meeting the sleep guidelines performed better on TEC than those who did not (mean difference [*MD*] = 1.41; *p* = 0.011). The difference in ToM performance between children meeting and not meeting the sleep and screen time guidelines approached significance (*MD* = 0.25; *p* = 0.052); There were no differences in TEC performance.	Meeting the sleep guidelines appeared to be more strongly associated with social-cognitive development than meeting the screen time guidelines. The combination of sufficient sleep and limited screen time might be associated with social-cognitive development in preschool children. Considering that higher levels of screen time are associated with shorter sleep durations, it may be hypothesized that screen time impacts social cognition through decreasing sleep duration.
Helm & Spencer (2019) [54]	7	*N* = 470 3–6 years (x̄ = 4.3, *SD* = 0.8) 46.2% Female 53.8% Male	USA	Cross-sectional	Sleep: actigraphy Screen: parent report Internalizing/ Externalizing: CBQ	Sleep (hrs/d): *M* = 10.24, *SD*: 0.68 Children who watch < 1 h of TV slept 14 min more than children who watch 1–3 h on an average weekday (F1, 251 = 11.11, *p* = 0.001). 36% of children had TVs in their bedrooms. Children without TVs in their bedrooms slept 30 min more at night compared to those with TV (F1, 299 = 43.75, *p* < 0.001). An ANCOVA revealed a significant main effect of bedroom TV presence for children’s negative affect (F1, 436 = 13.01, *p* < 0.01). Children with TVs in their bedrooms are rated higher on negative affect (*M* = 4.04, *SD* = 0.84) than children without TVs in their bedrooms (*M* = 3.71, *SD* = 0.90).	Children who watch little or no TV got more sleep overall and at night than children who watch more than hour of TV each day. The presence of TV in children’s bedrooms was associated with shorter sleep duration and poor sleep quality. Increased exposure to screens, especially before bedtime, may contribute to shorter sleep duration and poorer sleep quality in preschoolers, as well as higher negative affect scores.
Kahn et al. (2021) [55]	8	*N* = 145 3–6 years (x̄ = 4.9, *SD* = 0.7) 49.7% Female 50.3% Male	Israel	Cross-sectional	Sleep: actigraphy + parents report Screen: parents report Internalizing/ Externalizing: SDQ-P	Actigraphic sleep onset time, duration and efficiency were all associated with exposure to screens (*ps* < 0.005). Internalizing, externalizing and total SDQ-P scale scores were all associated with lower parent-reported sleep quality (*ps* < 0.005). Moderation analysis revealed a significant screen time x actigraphic sleep duration interaction effect for behavior problems (*b* = -0-03, *SD* = 0.02, *p* = 0.04). The overall model was significant (*R*^2^ = 0.17, F4, 140 = 7.01, *p* < 0.001). The model was also significant when evaluating externalizing problems alone *(R*^2^ = 0.15, F4, 140 = 6.26, *p* < 0.001).	Increased exposure to screens was associated with higher levels of behavioral problems only for children who slept less than 9.88 h per night on average. Sleep duration was found to moderate the relation between screen time and externalizing -but not internalizing- problems in preschoolers. Sleep quality and timing did not emerge as moderators of the link between screen time and behavior problems.
McNeill et al. (2020) [56]	8	*N* = 247 3–5 years (x̄ = 4.2, *SD* = 0.6) 40.1% Female 59.9% Male	Australia	Cross-sectional + 12-month longitudinal Prospective observational	Sleep: parent report Screen: parent report Cognition: EYT (executive functions) Externalizing/ Internalizing: SDQ	Sleep (hrs/d): *M* = 10.51, *SD* = 0.93; screen use (min/d): *M* = 140.30, *SD* = 82.17. Cross-sectional analyses suggested that meeting individual recommendations for sleep and screen use were not significantly associated with any of the executive function or externalizing and internalizing variables. No associations were found either when preschoolers met both guidelines. No longitudinal associations were observed for any of the behavioral or cognitive outcomes.	No associations were observed for meeting sleep and screen use guidelines with executive functions and psychological health in preschoolers. The large amounts of missing data resulted in smaller group comparisons and may have reduced variability of the data. This may have limited the ability to detect significant associations that are consistent with the current literature.
Mistry et al. (2007) [57]	6	*N* = 396 5.5 years 51.1% Female 48.9% Male	USA	Cross-sectional Prospective observational	Sleep: CBCL (sleep problems subscale) Screen: parents report Externalizing/ Internalizing: CBCL (emotionally reactive, anxious/depressed, attention problems and aggressive behavior subscales).	Linear regressions were performed to estimate the effect of TV exposure and having a TV in the child’s bedroom on children’s outcomes. Compared to preschoolers who were exposed to > 2 h of TV daily, those exposed to < 2 h showed lower scores in anxiety/ depression (β = 0.37, *p* < 0.01), attention problems (β = 0.37, *p* < 0.001), and aggressive behavior (β = 0.72, *p* < 0.05), but not with sleep problems. These differences disappeared when adjusting the model for covariates. Having a TV in the bedroom was associated with sleep problems (β = 0.22, *p* < 0.05) and lower emotional reactivity (β = -0.26, *p* < 0.01) in adjusted analyses.	Approximately 1 in 6 children viewed > 2 h of television daily. In addition, > 40% of preschoolers had a TV in their bedrooms. Having a TV in the bedroom likely leads to increased TV viewing at bedtime, thereby interfering with regular sleep patterns and decreasing the intensity with which children react to stimuli.
Nathanson y Beyens (2018) [58]	7	*N* = 402 3–5 years (x̄ = 4.0, *SD* = 0.8) 48% Female 52% Male	USA	Cross-sectional	Sleep: CSHQ + Parent report Screen: Parent report Externalizing/ Internalizing: ECB (Effortful Control Scale: negative affect, impulsivity, frustration, sadness, fear, activation…).	Sleep (hrs/d): *M* = 10.66, *SD* = 1.02; TV use (min/d): *M* = 228.15, *SD* = 158.42; Tablet use (min/d): *M* = 84.15, *SD* = 126.69; Handheld game player use (min/d): *M* = 35.34, *SD* = 83.61. Tablet use was negatively related to EC (β = -0.11. *p* = 0.029), but handheld game player use was positively related to Effortful control (EC) (β = 0.14, *p* = 0.004). Evening tablet use was associated with EC via: bedtimes, bedtime resistance, and sleep duration (*ps* < 0.05). Moderation analysis showed that: (1) the negative relation between tablet use and EC was only significant when children slept less (95% CI = 0.0001–0.0013); (2) the positive association between handheld game playing and EC was only significant when children slept longer (95% CI = 0.0001–0.0017).	Number of sleep hours moderated the relation between mobile media use and EC among preschoolers. Tablet time is negatively related to EC in preschoolers who get fewer hours of sleep. Handheld game playing is positively related to EC among preschoolers who get more hours of sleep. Sleep quality mediated the relation between evening tablet time and EC.
Nathanson y Fries (2015) [49]	7	*N* = 107 3–6 years (x̄ = 4.4, *SD* = 0.7) 49.5% Female 50.5% Male	USA	Cross-sectional	Sleep: parent report Screen time: screen time Cognition: ToM task Carlson Executive Function Task	TV use (min/d): *M* = 171.98, *SD* = 119.42 Total sleep time was related to ToM (*r* = 0.27, *p* < 0.01), nighttime television (*r* = -0.26, *p* < 0.01) and background TV exposure (*r* = -0.28, *p* < 0.01). Passive exposure was associated with shorter sleep duration (*r* = -0.28, *p* < 0.01). Mediation analysis showed an indirect effect of background TV on ToM through total sleep (95% CI = -0.00106 to -0.0001). Nighttime viewing had an indirect effect on ToM through sleep time (95% CI = -0-00448 to -0.0001).	Heavier background TV exposure and nighttime TV viewing were related to fewer hours of sleep, which, in turn, was related to weaker ToM. Sleep time did not mediate the relation between intentional TV viewing and Executive Functions. Correlation analyses confirmed that children’s sleep is negatively related to TV viewing, especially at night.
Séguin & Klimek (2016) [59]	5	*N* = 52 3–5 years (x̄ = 3.8, *SD* = 0.6) 48.1% Female 51.9% Male	Canada	Cross-sectional	Sleep: CSHQ Screen: parents report Externalizing/ Internalizing: PBQ	Pearson correlations showed significant negative correlations between amount of sleep and computer use (*r* = -0.38, *p* < 0.01), video game console use (*r* = -0.32, *p* < 0.05) and other electronic use (*r* = -0.33, *p <* 0.05). There were significant positive correlations between the amount of TV watched and all behavior dimensions: hostile-aggressive (*r* = 0.31, *p* < 0.05), anxious-fearful (*r* = 0.28, *p* < 0.05) and hyperactive-distractible (*r* = 0.33, *p* < 0.05). There was a positive correlation between the number of minutes a nighttime waking lasts and the anxious-fearful factor (*r* = 0.33, *p* < 0.05).	This study substantiated previous research that shows correlational relationships between electronic media use, sleep patterns and behavior.
Tso et al. (2016) [60]	8	*N* = 553 4–6 years (x̄ = 5.5, *SD* = 0.7) 52.1% Female 47.9% Male	China	Cross-sectional	Sleep: parents report ST: parent report Externalizing: CEDI (hyperactivity and inattention domain) Cognition: CEDI (language and cognitive ability domain)	70.5% of the sample slept 9–10 h/d and 43% used digital devices between 1–3 h/d. 24% slept ≤ 7 h/d and were considered to have sleep deprivation. Preschoolers who used electronic devices for more than 3 h slept 21 min less than those who used screens less than 1 h (*p* = 0.004). Compared to the reference group that slept 11–12 h, preschoolers who slept < 8 h/d scored lower in the language and cognitive domain, although with no significance (*OR* = 0.52, *p* = 0.06). Those who slept ≤ 7 h/d scored lower in this domain (*p* = 0.001). Compared to the reference group, those who slept < 8 h/d showed higher scores in hyperactivity and inattention (*p* = 0.03).	Children who used electronic devices for more than three hours per day had the shortest sleep durations. The findings suggest that exposure to screens might affect sleep onset and maintenance in preschoolers. Children with shorter sleep duration had lower scores in the language and cognitive domains. Sleep deprivation increased hyperactivity and inattention, which could impair executive functions, according to previous research.
Wu et al. (2017) [61]	7	*N* = 8900 3–6 years (x̄ = 4.4, *SD* = 0.9) 47.1% Female 52.9% Male	China	Cross-sectional	Sleep: parent report ST: parent report Externalizing/ Internalizing: SDQ	Percentages of children with ST ≥ 2 h/d and < 2 h/d were 42.7 and 57.3% respectively. Percentages of children with sleep duration < 9.15 h/d and ≥ 9.15 h/d were 57 and 43% respectively. Total SDQ scores were higher in children with longer ST (*MD* = 1.5, *p* < 0.001). Similar results were found for the relationship between SDQ total scores and shorter sleep duration. ST was positively correlated with the total SDQ score (*r* = 0.190, *p* < 0.001) Inversely, sleep duration was negatively correlated with the total SDQ scores (*r* = -0.079, *p* < 0.001 Children with more ST were significantly more likely to be at risk for behavioral problems compared with those with less ST (*OR* = 1.42, *p* < 0.001); the same was true for children with shorter sleep duration (*OR = 1.18, p* = 0.001). The simultaneous associations between ST and sleep and being at risk for behavioral problems were stronger (OR *= 1.72, p* = 0.001).	Preschool children with long ST and short sleep duration were significantly more likely to have behavioral problems, after controlling for potential confounding factors. In view of these results, it is important to limit children’s ST to no more than 1–2 h/d, as well as to regulate children’s sleep habits. Understanding the links between ST and sleep duration and behavior can pinpoint potential avenues for intervention.
Zhao et al. (2022) [62]	8	*N* = 3636 3–6 years (x̄ = 4.5, *SD* = 0.9) 45.8% Female 54.2% Male	China	Cross-sectional	Sleep: CSHQ ST: parents report Externalizing/ Internalizing: SDQ	Prevalence of sleep disturbances: 89.4%. Higher scores on hyperactivity and inattention, conduct problems, and emotional symptoms suggest higher CSHQ scores (*p* < 0.01 for all). The CSHQ scores were higher for those children with excessive ST compared to those with normal ST, especially for the subscales of sleep duration (*p* < 0.05), parasomnias (*p* < 0.01) and daytime sleepiness (*p* < 0.01).	The prevalence of sleep disturbances among preschoolers was high. The most common types were bedtime resistance and sleep anxiety. The results indicated that the characteristics of sleep disturbances in rural areas may differ from those in urban areas. There were correlations between children’s emotional/behavioral problems and sleep disturbances.
Zhao et al. (2018) [63]	7	*N* = 20,324 3–4 years (x̄ = 3.7, *SD* = 0.3) 47.3% Female 52.7% Male	China	Cross-sectional	Sleep: CSHQ ST: parent report Externalizing/ Internalizing: SDQ	Preschool children in the sample were exposed to 2.8 h/d of ST. with 78.6% exceeding 1 h/d and 53% exceeding 2 h/d. Compared with less than 1 h/d ST, the ORs for SDQ total difficulties were 1.2 (*p* < 0,001) for 1–2 h/d of screen time, 1.5 (*p* < 0.001) for 2–3 h/d, 1.6 (*p* < 0.001) for 3–4 h/d, and 2.1 (*p* < 0.001) for more than 4 h/d. The direct and total indirect effect of ST on total difficulties was, separately, 0.294 (*p* < 0.001) and 0.123 (*p* < 0.001). The effects of ST on total difficulties were mediated through sleep duration (0.5%).	Preschool children have an increasing risk of externalizing and internalizing problems with every additional hour of screen time. Sleep duration appears to be a significant mediator accounting for the effect of screen time on psychological well-being.

Note. SD: Standard Deviation; ST: Screen Time; CBCL: Child Behavior Checklist; TEC: Test of Emotional Comprehension; CBQ: Child Behavior Questionnaire; SDQ: Strength and Difficulty Questionnaire; EYT: Early Years Toolbox; ECB: Early Childhood Behavior Questionnaire; CSHQ: Children’s Sleep Habits Questionnaire; PBQ: Preschool Behavior Questionnaire; CEDI: Chinese Early Development Instrument

### Relationships between sleep and screen media

The studies made use of three primary mechanisms to explain the impact of screen use on sleep: direct exposure, passive exposure, and the presence of digital media in the preschoolers’ bedrooms. Direct exposure is consistently associated with shorter total sleep duration, both as measured by actigraphy [54] and by parent reports [49, 55, 59]. Positive associations are also found between prolonged exposure and increased daytime sleepiness [58, 59, 62], increased onset latency [55], increased bedtime resistance [58], and parasomnias [62]. Environmental or passive exposure is also associated with lower total sleep duration [49] and increased daytime sleepiness [59]. Finally, there is no consensus as to whether the presence of a TV in a child’s bedroom contributes to sleep disturbances.

### Relationships between Screen Media and behavioral and cognitive outcomes

In most of the studies, prolonged screen use is associated with a greater likelihood of internalizing and externalizing behaviors [52, 55, 59, 61, 63], with such associations being more consistent for externalizing behaviors. Additionally, Helm and Spencer’s [54] study reveals a main effect of the presence of TV in a child’s bedroom on negative affect; that is, children with a TV in their bedrooms scored higher on negative affect than those without. Furthermore, Nathanson and Beyens [58] report that the use of handheld video game consoles is associated with better scores on a self-control scale (negative affect, impulsivity, frustration…).

As regards cognitive performance, three studies show that excessive screen use is associated with lower scores on executive functioning [49, 58], Theory of Mind (ToM) tasks [49], and attention [57]. However, two other studies find no such associations [53, 56]. Thus, there is no clear evidence supporting relationships between screen time and poorer performance on cognitive tasks. Furthermore, passive TV exposure and the presence of a TV in a child’s bedroom correlates negatively with scores on Theory of Mind and Executive Functions [49].

### Relationships between Sleep and behavioral and cognitive outcomes

Shorter sleep duration than recommended is significantly related to internalizing and externalizing problems in various studies [60–63], although these associations are less clear in the case of internalizing behaviors. Wu et al. [61], for example, find that children whose sleep duration was lower than the group mean presented a greater risk of behavioral problems and hyperactivity, but report no significant increase in the risk of emotional symptoms. Meanwhile, Kahn et al. [55] found significant relationships between parent-reported sleep quality and higher internalizing and externalizing scores, but no such associations were found for sleep duration, measured both by actigraph and by parental report. Finally, Séguin and Klimek [59] only observed a positive relationship between the number and duration of nighttime awakenings and symptoms of anxiety or fear.

The relationship between sleep and cognitive variables has been the subject of less research, with inconclusive results. While Nathanson and Fries [49] report that longer total sleep duration significantly correlates with better performance on Theory of Mind, Cliff et al. [53] find no such association. In no case are differences in executive function scores found depending on sleep duration and sleep quality [49, 56]. Finally, in the study by Tso et al. [60], the group classified as “sleep-deprived” (children who slept ≤ 7 h per day during the assessment week) scored significantly lower in the cognitive and language domain compared to the reference group that slept the recommended number of hours.

### Sleep as Mediator or Moderator

Some of the studies examined whether sleep acts as a moderating or mediating factor between screen use and behavioral and/or cognitive problems.

The work by Kahn et al. [55] uses sleep duration measured with an actigraph as a mediating variable between screen use and the appearance of internalizing and externalizing behaviors. Screen use explained 17% of the variance in behavioral scores when sleep was included as a mediator. Their regression slope analysis showed that greater exposure to screens was associated with behavioral problems in children with low sleep duration (this group of participants showed an average of 9.24 h of sleep per day). However, screen use time was not associated with higher levels of behavioral problems in children whose sleep duration was moderate or high (with sleep averages of 9.85 h/day and 10.46 h/day respectively).

Zhao et al. [63] conducted a similar analysis. In their study, the direct and indirect effects of screen use on total internalizing and externalizing scores were separately 0.294 (95% CI: 0.261, 0. 328; *p* < 0.001) and 0.123 (95% CI 0.112, 0.135; *p* < 0.001), respectively, which accounted for 0.417 as the total effect. These effects were mediated by sleep duration (0.5%).

Furthermore, the findings of Nathanson and Beyens [58] suggest that afternoon/evening tablet use is significantly associated with worse scores on attention and impulse control when bedtime (95% CI = -0.0015, -0.0002)]), bedtime resistance (95% CI = -0.011, -0.00001) and nighttime sleep duration (95% CI = -0.0014, 0–0002) are used as mediators. Additionally, the relationship between tablet use and scores on attention and inhibition was only significant when the child slept less (Coef. = 0.0007, 95% CI = 0.0001, 0.0013). The cut-off point for sleep duration was less than 10.61hs/day. In contrast, a positive association was found between the use of video game consoles and better attention and inhibition scores, but this was only significant for children who slept longer (Coef. = 0.0009, 95% CI = 0.0001, 0.0017). Sleep duration was shown to be a significant moderator when children slept more than 10.42 h/day.

Finally, Nathanson and Fries [49] report two main findings: (a) an indirect effect of background TV on Theory of Mind scores through lower sleep duration; (b) sleep duration mediates the relationship between afternoon/evening TV viewing and ToM performance, with the results of the analysis being significant.

## Discussion

The aim of this systematic review was to provide an overview of the state of the art on how excessive screen use affects sleep-related aspects in preschoolers, which, in turn, influences behavioral and cognitive aspects.

The results of the review suggest that less than half of the 3- to 6-year olds meet health authorities’ recommendations for exposure to screens (less than an hour a day), which coincides with the findings of a recent meta-analysis [64]. This prolonged use may affect sleep parameters through the following: both active and passive exposure, especially at night; increased presence of digital devices in a child’s environment; and displacement of other activities that may favor sleep onset [49, 54, 55, 58, 62, 65]. These patterns of digital device use are consistently associated with (a) a reduction in the total amount of sleep, which is in line with previous research (e.g., [61, 62, 66]); and (b) worse sleep patterns, observable in aspects such as increased bedtime resistance, more nighttime awakenings and daytime sleepiness, results which also align with those of other studies (e.g., [63, 64, 67, 68]). Therefore, our findings point towards a multifaceted impact of screen use on preschoolers’ sleep, such that those presenting greater exposure to screens are the most likely to have shorter duration and poorer quality of sleep.

Furthermore, small to moderate effects of screen use on behavioral and cognitive problems were found [52, 55, 62]. Exposure to more than one hour of screen time in preschoolers appears to be associated with a greater risk of an onset of emotional difficulties, increased negative affect and anxiety, as well as aggressive, hostile, or hyperactive behaviors. Additionally, the data show more robust associations between screen time and externalizing behaviors compared to internalizing behaviors, coinciding with previous research [69]. As regards the relationship between screen use and cognitive variables, drawing conclusions is complicated by the lack of studies. However, small to moderate negative associations were found between increased screen exposure and performance in attention, executive functioning, and theory of mind [49, 57, 58]. Additionally, Nathanson and Beyens [58] found that moderate use of video game consoles may improve preschoolers’ attention. These relationships might be influenced by factors such as the multimedia content children engage with or the time of day when they are most exposed [69]. The results of the present review are consistent with the current literature, which reports inconclusive findings on the impact of digital devices on younger children’s psychological and cognitive well-being [70, 71].

Regarding the relationship between sleep and behavioral problems, broadly speaking, the results suggest that lower sleep duration and quality leads to a greater likelihood of externalizing and internalizing problems [55, 59–63]. This relationship appears to be more consistent for externalizing problems, arguably because these types of behavioral problems tend to be more common in the preschool years and decrease with age, while internalizing behaviors typically follow a contrasting developmental trajectory [72]. Moreover, although there seems to be a relationship between sleep disorders and certain cognitive functioning variables such as Theory of Mind [49], there is a lack of agreement between the studies included in the review.

Finally, this review was intended to determine whether sleep duration and sleep quality interact with screen time to predict greater behavioral or cognitive problems. Increased screen exposure is associated with higher scores on behavioral problems (primarily externalizing) and lower scores on cognitive performance (attention, EF, and ToM) when sleep duration is short [49, 58, 63]. Furthermore, the results appear to show a greater influence of factors such as nighttime screen use, or passive and prolonged exposure, rather than total active use time. Although a substantial number of studies report associations between screen use and psychopathology in children, the size of these relationships varies from moderate to small [73], suggesting that other factors may enhance or bolster the consequences of screen use. In this respect, some researchers have suggested that sleep is a bioregulatory system that plays a role in children’s ability to self-regulate emotions, behavior, and thought; in other words, sleep may both protect from, and contribute to, the occurrence of childhood psychopathology depending on the context, with prolonged exposure to screens being a risk factor for sleep impairment [74].

### Study limitations and future directions

Firstly, this systematic review was affected by the heterogeneous nature of the studies included. This heterogeneity is evident in both the great variability of the sample sizes and in the diverse definitions and forms of measurement of the variables under study. In this sense, it is worth highlighting the absence of standardized questionnaires for assessing screen use in preschool population, which might have impacted the validity of the results. Similarly, much of the research focuses on the use of tests and sleep diaries to be completed by parents, while the use of objective measures, such as actigraphy, is less common. Additionally, the authors of the articles included use seven different questionnaires to measure behavioral and cognitive variables, with these mostly being completed by the children’s parents, who are familiar with both their children’s sleep status and their screen use, which may enhance the risk of bias.

Secondly, the current state of the literature makes it difficult to analyze specific aspects of screen use (e.g., time of use, passive exposure…), sleep (e.g., duration, bedtime resistance, onset latency…), and behavior and cognition, mainly due to the great variation in the designs and statistical analyses implemented. The quality and comparability of findings could be improved by focusing research on specific aspects of both exposure and outcome variables. Additionally, different aspects of screen use or sleep cannot always be assumed to have the same effect on behavioral and cognitive factors.

Thirdly, it is worth noting that the vast majority of studies are cross-sectional, and are thus unable to establish a causal relationship between screen exposure or sleep and behavioral and/or cognitive aspects. The literature suggests that high levels of screen use affect both sleep duration and quality, and that this, in turn, has an impact on behavior and cognition in preschoolers. However, it is plausible that other factors may explain some of these relationships, such as, for example, parent-child communication. Further longitudinal studies are required to determine the direction of the impact of these variables on each other.

Finally, few of the works include mediation or moderation analysis among the statistical methods used. This makes it difficult to make comparisons between studies and to draw clear conclusions on the mediating or moderating role of sleep in the relationship between screen use and behavioral and cognitive disorders.

As future research, we propose delving into the associations between specific aspects of screen use, sleep, and behavioral and cognitive measures, using longitudinal studies with sufficient statistical power. In addition, the use of objective measures for sleep measurement would be of interest, as would be the assessment of behavioral or cognitive outcomes by a researcher uninformed of the sample’s sleep status or screen use. It would be useful to create a strictly defined protocol that would allow criteria to be unified when selecting and measuring the different variables, thus facilitating the comparison and interpretation of the results obtained in studies.

## Conclusions

The current literature appears to suggest that screen use and sleep are related to behavioral and cognitive development in preschool years, but further research is needed to determine the scope and potential of these relationships. Studies in this area could assist in the creation of policies and general recommendations for parents, educators, physicians and other professionals. Additionally, deeper knowledge of how digital devices are used from early childhood and how they affect sleep and intra-individual aspects would allow for the development of effective early interventions on such overuse.

## References

[1] Lissak G (2018) Adverse physiological and psychological effects of screen time on children and adolescents: literature review and case study. Environ Res 164:149–157 29499467 10.1016/j.envres.2018.01.015

[2] Moorman JD, Morgan P, Adams TL (2019) The implications of screen media use for the sleep behavior of children ages 0–5: a systematic review of the literature. Curr Sleep Med Rep 5(3):164–172

[3] Basile C, Gigliotti F, Cesario S, Bruni O (2021) The relation between sleep and neurocognitive development in infancy and early childhood: a neuroscience perspective. Adv Child Dev Behav 60:9–27 33641802 10.1016/bs.acdb.2020.11.003

[4] Bernier A, Cimon-Paquet C, Tétreault É (2021) Sleep development in preschool predicts executive functioning in early elementary school. In: Berger SE, Harbourne RT, Scher A, editors. Advances in Child Development and Behavior [Internet]. Elsevier; pp. 159–78. 10.1016/bs.acdb.2020.08.005 10.1016/bs.acdb.2020.08.005 33641792

[5] Nieto M, Motos B, Navarro B, Jimeno MV, Fernández-Aguilar L, Ros L et al (2022) Relation between nighttime sleep duration and executive functioning in a nonclinical sample of preschool children. Scand J Psychol 10.1111/sjop.12801 35286756

[6] Hoyniak CP, Bates JE, McQuillan ME, Staples AD, Petersen IT, Rudasill KM et al (2020) Sleep across early childhood: implications for internalizing and externalizing problems, socioemotional skills, and cognitive and academic abilities in preschool. J Child Psychol Psychiatry 61(10):1080–1091 32173864 10.1111/jcpp.13225 PMC7812691

[7] Williams KE, Berthelsen D, Walker S, Nicholson JM (2017) A developmental cascade model of behavioral sleep problems and emotional and attentional self-regulation across early childhood. Behav Sleep Med 15(1):1–21 26619760 10.1080/15402002.2015.1065410

[8] Chen X, Beydoun MA, Wang Y (2008) Is sleep duration associated with childhood obesity? A systematic review and meta-analysis. Obesity 16(2):265 18239632 10.1038/oby.2007.63

[9] Meltzer LJ, Plaufcan MR, Thomas JH, Mindell JA (2014) Sleep problems and sleep disorders in pediatric primary care: treatment recommendations, persistence, and health care utilization. J Clin Sleep Med 10(4):421–426 24733988 10.5664/jcsm.3620 PMC3960385

[10] Sadeh A, Tikotzky L, Scher A (2010) Parenting and infant sleep. Sleep Med Rev 14(2):89–96 19631566 10.1016/j.smrv.2009.05.003

[11] Hiscock H, Bayer J, Gold L, Hampton A, Ukoumunne OC, Wake M (2007) Improving infant sleep and maternal mental health: a cluster randomised trial. Arch Dis Child 92(11):952–958 17158146 10.1136/adc.2006.099812 PMC2083609

[12] Meltzer LJ, Mindell JA (2007) Relationship between child sleep disturbances and maternal sleep, mood, and parenting stress: a pilot study. J Fam Psychol 21(1):67 17371111 10.1037/0893-3200.21.1.67

[13] Buysse DJ (2014) Sleep health: can we define it? Does it Matter? Sleep 37(1):9–17 24470692 10.5665/sleep.3298 PMC3902880

[14] Meltzer LJ, Williamson AA, Mindell JA (2021) Pediatric sleep health: it matters, and so does how we define it. Sleep Med Rev 57:1–13 10.1016/j.smrv.2021.101425 PMC9067252 33601324

[15] Reynaud E, Vecchierini M, Heude B, Charles M, Plancoulaine S (2018) Sleep and its relation to cognition and behaviour in preschool-aged children of the general population: a systematic review. J Sleep Res 27(3):e12636 29164715 10.1111/jsr.12636

[16] Astill RG, Van der Heijden KB, Van IJzendoorn MH, Van Someren EJ (2012) Sleep, cognition, and behavioral problems in school-age children: a century of research meta-analyzed. Psychol Bull 138(6):1109 22545685 10.1037/a0028204

[17] Arts J, Gubbels JS, Verhoeff AP, Chinapaw MJM, Lettink A, Altenburg TM (2022) A systematic review of proxy-report questionnaires assessing physical activity, sedentary behavior and/or sleep in young children (aged 0–5 years). Int J Behav Nutr Phys Act 19(1):18 35164783 10.1186/s12966-022-01251-x PMC8845346

[18] Sadeh A, Sharkey M, Carskadon MA (1994) Activity-based sleep-wake identification: an empirical test of methodological issues. Sleep 17(3):201–207 7939118 10.1093/sleep/17.3.201

[19] Fabbri M, Beracci A, Martoni M, Meneo D, Tonetti L, Natale V (2021) Measuring subjective sleep quality: a review. Int J Environ Res Public Health 18(3):1082 33530453 10.3390/ijerph18031082 PMC7908437

[20] Beck SE, Marcus CL (2009) Pediatric Polysomnography. Sleep Med Clin 4(3):393–406 20161110 10.1016/j.jsmc.2009.04.007 PMC2739664

[21] Coyle-Asbil HJ, Breau B, Ma DW, Haines J, Vallis LA (2020) Examining the effects of applying ActiGraph low-frequency extension feature to analyze the sleeping behaviours of preschool-aged children. Appl Physiol Nutr Metab 45(12):1396–1399 32780964 10.1139/apnm-2019-0969

[22] Smith C, Galland B, Taylor R, Meredith-Jones K (2020) ActiGraph GT3X + and actical wrist and hip worn accelerometers for sleep and wake indices in young children using an automated algorithm: validation with polysomnography. Front Psychiatry 10:958 31992999 10.3389/fpsyt.2019.00958 PMC6970953

[23] Sadeh A, De Marcas G, Guri Y, Berger A, Tikotzky L, Bar-Haim Y (2015) Infant sleep predicts attention regulation and behavior problems at 3–4 years of age. Dev Neuropsychol 40(3):122–137 26151611 10.1080/87565641.2014.973498

[24] Bathory E, Tomopoulos S (2017) Sleep regulation, physiology and development, sleep duration and patterns, and sleep hygiene in infants, toddlers, and preschool-age children. Curr Probl Pediatr Adolesc Health Care 47(2):29–42 28117135 10.1016/j.cppeds.2016.12.001

[25] Page JM, Wakschlag LS, Norton ES (2021) Nonrapid eye movement sleep characteristics and relations with motor, memory, and cognitive ability from infancy to preadolescence. Dev Psychobiol 63(8):1–18 10.1002/dev.22202 PMC8898567 34813099

[26] Galland BC, Short MA, Terrill P, Rigney G, Haszard JJ, Coussens S et al (2018) Establishing normal values for pediatric nighttime sleep measured by actigraphy: a systematic review and meta-analysis. Sleep 41(4):zsy017 10.1093/sleep/zsy017 29590464

[27] Pin G, Sampedro M (2018) Fisiología Del sueño y sus trastornos. Ontogenia Y evolución del sueño a lo largo de la etapa pediátrica. Relación Del sueño con la alumentación. Clasificación De Los problemas y trastornos del sueño. Pediatría Integr 22(8):358–371

[28] Ohayon M, Wickwire EM, Hirshkowitz M, Albert SM, Avidan A, Daly FJ et al (2017) National Sleep Foundation’s sleep quality recommendations: first report. Sleep Health 3(1):6–19 28346153 10.1016/j.sleh.2016.11.006

[29] American Academy of Pediatrics (2013) Children, adolescents and the media. Pediatrics 132(5):958–961 28448255 10.1542/peds.2013-2656

[30] World Health Organization (2019) To grow up healthy, children need to sit less and play more. World Health Organ WHO News Httpswww Who Intnewsitem24-04-2019–Grow–Healthy-Child-Need–Sit—Play-More

[31] Lee S, Matsumori K, Nishimura K, Nishimura Y, Ikeda Y, Eto T et al (2018) Melatonin suppression and sleepiness in children exposed to blue-enriched white LED lighting at night. Physiol Rep 6(24):e13942 30556352 10.14814/phy2.13942 PMC6295443

[32] Hartstein LE, Behn CD, Akacem LD, Stack N, Wright KP Jr, LeBourgeois MK (2022) High sensitivity of melatonin suppression response to evening light in preschool-aged children. J Pineal Res 72(2):e12780 34997782 10.1111/jpi.12780 PMC8933063

[33] Cheung CH, Bedford R, De Saez IR, Karmiloff-Smith A, Smith TJ (2017) Daily touchscreen use in infants and toddlers is associated with reduced sleep and delayed sleep onset. Sci Rep 7(1):1–7 28406474 10.1038/srep46104 PMC5390665

[34] Nathanson AI (2021) Sleep and technology in early childhood. Child Adolesc Psychiatr Clin 30(1):15–26 10.1016/j.chc.2020.08.008 33223059

[35] Garrison MM, Liekweg K, Christakis DA (2011) Media use and child sleep: the impact of content, timing, and environment. Pediatrics 128(1):29–35 21708803 10.1542/peds.2010-3304 PMC3124101

[36] Magee CA, Lee JK, Vella SA (2014) Bidirectional relationships between sleep duration and screen time in early childhood. JAMA Pediatr 168(5):465–470 24589672 10.1001/jamapediatrics.2013.4183

[37] Williamson AA, Davenport M, Cicalese O, Mindell JA (2021) Sleep problems, cumulative risks, and psychological functioning in early childhood. J Pediatr Psychol 46(7):878–890 33738501 10.1093/jpepsy/jsab022 PMC8357222

[38] Marcone R, Affuso G, Borrone A (2020) Parenting styles and children’s internalizing-externalizing behavior: the mediating role of behavioral regulation. Curr Psychol 39(1):13–24

[39] Scharf RJ, Demmer RT, Silver EJ, Stein RE (2013) Nighttime sleep duration and externalizing behaviors of preschool children. J Dev Behav Pediatr 34(6):384–391 23838583 10.1097/DBP.0b013e31829a7a0d

[40] Hall W, Scher A, Zaidman-Zait A, Espezel H, Warnock F (2012) A community‐based study of sleep and behaviour problems in 12‐to 36‐month‐old children. Child Care Health Dev 38(3):379–389 21651607 10.1111/j.1365-2214.2011.01252.x

[41] Zaidman-Zait A, Hall WA (2015) Children’s night waking among toddlers: relationships with mothers’ and fathers’ parenting approaches and children’s behavioural difficulties. J Adv Nurs 71(7):1639–1649 25689874 10.1111/jan.12636

[42] Bélanger MÈ, Bernier A, Simard V, Desrosiers K, Carrier J (2018) Sleeping toward behavioral regulation: relations between sleep and externalizing symptoms in toddlers and preschoolers. J Clin Child Adolesc Psychol 47(3):366–373 26605811 10.1080/15374416.2015.1079782

[43] Hatzinger M, Brand S, Perren S, Stadelmann S, von Wyl A, von Klitzing K et al (2010) Sleep actigraphy pattern and behavioral/emotional difficulties in kindergarten children: association with hypothalamic-pituitary-adrenocortical (HPA) activity. J Psychiatr Res 44(4):253–261 19762039 10.1016/j.jpsychires.2009.08.012

[44] Anders T, Iosif AM, Schwichtenberg A, Tang K, Goodlin-Jones B (2012) Sleep and daytime functioning: a short-term longitudinal study of three preschool-age comparison groups. Am J Intellect Dev Disabil 117(4):275–290 22809074 10.1352/1944-7558-117.4.275 PMC3500838

[45] Nieto M, Ros L, Ricarte JJ, Latorre JM (2018) The role of executive functions in accessing specific autobiographical memories in 3- to 6- year-olds. Early Child Res Q 43:23–32

[46] Reynaud E, Forhan A, Heude B, Charles MA, Plancoulaine S (2021) Night-sleep duration trajectories and behavior in preschoolers: results from a prospective birth cohort study. Behav Sleep Med 19(4):445–457 32497438 10.1080/15402002.2020.1773467

[47] Short MA, Blunden S, Rigney G, Matricciani L, Coussens S, Reynolds CM et al (2018) Cognition and objectively measured sleep duration in children: a systematic review and meta-analysis. Sleep Health 4(3):292–300 29776624 10.1016/j.sleh.2018.02.004

[48] Olini H, Huber R (2014) Ageing and sleep: Sleep in all stages of human development. In: Bassetti C, Huber R, editors. ESRS sleep medicine textbook [Internet]. European Sleep Research Society; pp. 73–82. http://www.esrs.eu/esrs/sleep-medicine-textbook.html

[49] Nathanson AI, Fries PT (2015) Television exposure, sleep time, and neuropsychological function among preschoolers. Media Psychol 17(3):237–261

[50] Peled M, Scher A (2021) The contribution of good sleep to working memory in preschool: A matter of sleep quality or duration? Adv Child Dev Behav 60:85–110 33641801 10.1016/bs.acdb.2020.11.001

[51] Wells G, Brodsky L, O’Connell D, Shea B, Henry D, Mayank S et al (2003) An evaluation of the Newcastle Ottawa Scale: an assessment tool for evaluating the quality of non-randomized studies. In Book of Abstracts; p. 26

[52] Carson V, Ezeugwu VE, Tamana SK, Chikuma J, Lefebvre DL, Azad MB et al (2019) Associations between meeting the Canadian 24-Hour Movement guidelines for the early years and behavioral and emotional problems among 3-year-olds. J Sci Med Sport 22(7):797–802 30655179 10.1016/j.jsams.2019.01.003

[53] Cliff DP, McNeill J, Vella SA, Howard SJ, Santos R, Batterham M et al (2017) Adherence to 24-hour movement guidelines for the early years and associations with social-cognitive development among Australian preschool children. BMC Public Health 17(5):207–215 29219104 10.1186/s12889-017-4858-7 PMC5773906

[54] Helm AF, Spencer RM (2019) Television use and its effects on sleep in early childhood. Sleep Health 5(3):241–247 30987948 10.1016/j.sleh.2019.02.009 PMC6581597

[55] Kahn M, Schnabel O, Gradisar M, Rozen GS, Slone M, Atzaba-Poria N et al (2021) Sleep, screen time and behaviour problems in preschool children: an actigraphy study. Eur Child Adolesc Psychiatry 30(11):1793–1802 33006004 10.1007/s00787-020-01654-w

[56] McNeill J, Howard SJ, Vella SA, Cliff DP (2020) Compliance with the 24-Hour movement guidelines for the early years: cross-sectional and longitudinal associations with executive function and psychosocial health in preschool children. J Sci Med Sport 23(9):846–853 32360244 10.1016/j.jsams.2020.02.011

[57] Mistry KB, Minkovitz CS, Strobino DM, Borzekowski DL (2007) Children’s television exposure and behavioral and social outcomes at 5.5 years: does timing of exposure matter? Pediatrics 120(4):762–769 17908763 10.1542/peds.2006-3573

[58] Nathanson AI, Beyens I (2018) The role of sleep in the relation between young children’s mobile media use and effortful control. Br J Dev Psychol 36(1):1–21 28792067 10.1111/bjdp.12196

[59] Séguin D, Klimek V (2016) Just five more minutes please: electronic media use, sleep and behaviour in young children. Early Child Dev Care 186(6):981–1000

[60] Tso W, Rao N, Jiang F, Li AM, Lee S, lun, Ho FK et al (2016) wing,. Sleep duration and school readiness of Chinese preschool children. J Pediatr. 169:266–71 10.1016/j.jpeds.2015.10.064 26608085

[61] Wu X, Tao S, Rutayisire E, Chen Y, Huang K, Tao F (2017) The relationship between screen time, nighttime sleep duration, and behavioural problems in preschool children in China. Eur Child Adolesc Psychiatry 26(5):541–548 27822641 10.1007/s00787-016-0912-8

[62] Zhao T, Xuan K, Liu H, Chen X, Qu G, Wu Y et al (2022) Sleep disturbances and correlates among a sample of preschool children in rural China. Sleep Biol Rhythms 20(1):123–136 38469069 10.1007/s41105-021-00348-3 PMC10900050

[63] Zhao J, Zhang Y, Jiang F, Ip P, Ho FKW, Zhang Y et al (2018) Excessive screen time and psychosocial well-being: the mediating role of body mass index, sleep duration, and parent-child interaction. J Pediatr 202:157–162 30100232 10.1016/j.jpeds.2018.06.029

[64] McArthur BA, Volkova V, Tomopoulos S, Madigan S (2022) Global prevalence of meeting screen time guidelines among children 5 years and younger: a systematic review and meta-analysis. JAMA Pediatr 10.1001/jamapediatrics.2021.6386 PMC8845032 35157028

[65] Chonchaiya W, Wilaisakditipakorn T, Vijakkhana N, Pruksananonda C (2017) Background media exposure prolongs nighttime sleep latency in Thai infants. Pediatr Res 81(2):322–328 27814342 10.1038/pr.2016.228

[66] Twenge JM, Hisler GC, Krizan Z (2019) Associations between screen time and sleep duration are primarily driven by portable electronic devices: evidence from a population-based study of US children ages 0–17. Sleep Med 56:211–218 30639033 10.1016/j.sleep.2018.11.009

[67] Brambilla P, Giussani M, Pasinato A, Venturelli L, Privitera F, del Miraglia E et al (2017) Sleep habits and pattern in 1–14 years old children and relationship with video devices use and evening and night child activities. Ital J Pediatr 43(1):1–11 28257638 10.1186/s13052-016-0324-x PMC5347825

[68] Paavonen EJ, Pennonen M, Roine M, Valkonen S, Lahikainen AR (2006) TV exposure associated with sleep disturbances in 5-to 6‐year‐old children. J Sleep Res 15(2):154–161 16704570 10.1111/j.1365-2869.2006.00525.x

[69] Xie G, Deng Q, Cao J, Chang Q (2020) Digital screen time and its effect on preschoolers’ behavior in China: results from a cross-sectional study. Ital J Pediatr 46(1):1–7 31973770 10.1186/s13052-020-0776-x PMC6979375

[70] Eirich R, McArthur BA, Anhorn C, McGuinness C, Christakis DA, Madigan S (2022) Association of screen time with internalizing and externalizing behavior problems in children 12 years or younger: a systematic review and meta-analysis. JAMA Psychiatry 10.1001/jamapsychiatry.2022.0155 PMC8928099 35293954

[71] Mallawaarachchi SR, Anglim J, Hooley M, Horwood S (2022) Associations of smartphone and tablet use in early childhood with psychosocial, cognitive and sleep factors: a systematic review and meta-analysis. Early Child Res Q 60:13–33

[72] Bongers IL, Koot HM, Van der Ende J, Verhulst FC (2003) The normative development of child and adolescent problem behavior. J Abnorm Psychol 112(2):179 12784827 10.1037/0021-843x.112.2.179

[73] Inoue S, Yorifuji T, Kato T, Sanada S, Kawachi I (2016) Children’s media use and self-regulation behavior: longitudinal associations in a nationwide Japanese study. Matern Child Health J 20(10):2084–2099 27334636 10.1007/s10995-016-2031-z

[74] Cremone A, de Jong DM, Kurdziel LB, Desrochers P, Sayer A, LeBourgeois MK et al (2018) Sleep tight, act right: negative affect, sleep and behavior problems during early childhood. Child Dev 89(2):e42–59 28129449 10.1111/cdev.12717 PMC5529275

